# 679. Burn Therapy Guideline for Treatment Timing and Staffing Allocation: Driving Outcomes, Efficiency, and FTE Justification

**DOI:** 10.1093/jbcr/irag033.560

**Published:** 2026-04-06

**Authors:** Lindsay DeSantis, Samantha Allbritton, Laurin Proctor, Benson Pulikkottil, Wojciech Przylecki, Lily Daniali, Ryan Endress, Heather Missimer, Elizabeth Tzetzo, Michelle Oliveti, Michelle Pankey, Nicole Henry

**Affiliations:** HCA HealthONE Swedish, Englewood, CO; HCA HealthONE Swedish, Englewood, CO; HCA HealthONE Swedish, Englewood, CO; HCA HealthONE Swedish, Englewood, CO; HCA HealthONE Swedish, Englewood, CO; HCA HealthONE Swedish, Englewood, CO; HCA HealthONE Swedish, Englewood, CO; HCA HealthONE Swedish, Englewood, CO; HCA HealthONE Swedish, Englewood, CO; HCA HealthONE Swedish, Englewood, CO; HCA HealthONE Swedish, Englewood, CO; HCA HealthONE Swedish, Englewood, CO

## Abstract

**Introduction:**

Burn injuries require individualized rehabilitation intensity to optimize function, prevent complications, and ensure recovery. A literature review demonstrates limited discussion of therapy allocation practices specific to burn populations. This guideline standardizes acute therapy treatment times and staff utilization through a tiered model based on total body surface area (TBSA) burned.

**Methods:**

Using literature review, workflow analysis, and expert consensus, patients were stratified into four tiers by TBSA involvement and clinical needs. Tier 1 (< 10% TBSA) and Tier 2 (10-19% TBSA) emphasize education, mobility, and home exercise. Tier 3 (20-49% TBSA) and Tier 4 (> 50% TBSA) include structured therapy 4-7 days per week, with prescribed treatment time set at 3 minutes per TBSA percentage per day, distributed across physical, occupational, and speech therapy disciplines.

**Results:**

The guideline establishes clear benchmarks for daily therapy duration: e.g., 20% TBSA = 60 minutes, 50% TBSA = 150 minutes, and 100% TBSA = 300 minutes per day. This system enhances predictability in staff scheduling, aligns therapy intensity with injury burden, and supports equitable resource distribution. With this structured model, the Burn Therapy Team and Service Leadership were able to demonstrate increased therapist productivity by 167% and an increase of therapy minutes by 150% within a four-month period. Further integration into burn service workflows allowed earlier mobilization, supported functional independence, and promoted consistency across providers and the patient population.

**Conclusions:**

Implementation of a standardized, TBSA-based therapy allocation model provides a reproducible method to align treatment intensity with injury severity while optimizing staff utilization. The utilization of this guideline successfully increased therapist productivity and increased therapy treatment minutes per patient.

**Applicability of Research to Practice:**

This evidence-informed guideline offers a scalable framework for improving outcomes in acute burn rehabilitation and can serve as a model for other high-acuity care settings.

**Funding for the study:**

N/A.

